# Multi-omics analysis reveals key genes associated with clear cell renal cell carcinoma

**DOI:** 10.7717/peerj.21057

**Published:** 2026-04-21

**Authors:** Xiaochen Chen, Tian Han, Mingwei Zhan, Xi Zhang, Weizhuo Wang, Ke Wu

**Affiliations:** 1 Radiation Oncology, The Affiliated Suzhou Hospital of Nanjing Medical University, Suzhou, Jiangsu, China; 2 Urology, The First Affiliated Hospital of Nanjing Medical University, Nanjing, Jiangsu, China; 3 Urology, Nanjing University, Nanjing, Jiangsu, China; 4 Urology, The Affiliated Suzhou Hospital of Nanjing Medical University, Suzhou, China

**Keywords:** Single cell sequencing, Clear cell renal cell carcinomas, Mendelian randomization analysis, Immune microenvironment, Biomarker

## Abstract

**Background:**

The most common type of kidney cancer is ccRCC (Clear Cell Renal Cell Carcinoma). Existing research has shown that when kidney cancer progresses from T2 to T3 or T4 stage, the treatment options and prognosis significantly differ. The aim of this study is to identify prognosis-related genes in ccRCC using single-cell data and Mendelian randomization analysis.

**Methods:**

Single-cell RNA sequencing (scRNA-seq) data from six ccRCC patients (GSE156632) were analyzed in R. After quality control and normalization, cells were embedded, clustered, and annotated to define major cell types. Cell–cell signaling networks were inferred using CellChat. Stage-related co-expression programs were identified using high-dimensional weighted gene co-expression network analysis (hdWGCNA), and modules enriched in advanced disease were prioritized. To connect expression patterns with germline variation, we obtained expression quantitative trait loci (eQTL) summary statistics from the UK Biobank and performed two-sample Mendelian randomization using the ukb-b-1316 exposure dataset. For clinical validation, bulk RNA-seq and survival data from The Cancer Genome Atlas (TCGA) were incorporated, and candidate genes were evaluated using univariable Cox proportional hazards models.

**Results:**

hdWGCNA identified three modules, from which we selected 150 genes. eQTL combined with Mendelian randomization analysis revealed a potential causal relationship between RBP5, PRDX2, GTSF1, BSG, and COX14 and tumorigenesis. Further TCGA data analysis indicated that PRDX2 might serve as a protective factor in ccRCC, consistent with the Mendelian randomization results. Furthermore, its expression exhibited a gradual decline with tumor stage progression. Kaplan-Meier(KM) curves demonstrated better prognosis for patients with high PRDX2 expression. Cell communication analysis revealed more frequent communication between cells with reduced PRDX2 expression and vascular endothelial cells. Moreover, the experiments demonstrate that the migration and proliferation abilities of ccRCC cells are enhanced after knocking down PRDX2.

**Conclusion:**

These results suggest that PRDX2 may play a role in the progression of ccRCC and could potentially serve as a prognostic factor and therapeutic target for ccRCC.

## Introduction

Renal cell carcinoma is a malignant neoplasm arising from renal parenchymal cells and accounts for most adult kidney cancers. Among its histological categories, clear cell renal cell carcinoma (ccRCC) is the predominant subtype, comprising roughly 85–90% of cases (Bahadoram et al., 2022). Tumor stage is assigned according to size and the extent of local invasion. Lesions confined to the kidney are designated T1–T2 (Stolpa, Strek-Cholewinska & Mizia-Malarz, 2022), whereas extension into the renal vein, inferior vena cava, adrenal gland, perinephric fat, or renal fascia classifies tumors as T3–T4 (Hashmi & Limaiem, 2023). This stage transition is clinically meaningful: while localized disease is commonly managed with partial or radical nephrectomy, invasive tumors often require more aggressive strategies and are associated with poorer survival (Williamson, Taneja & Cheng, 2019).

Metastatic spread and local infiltration of ccRCC are strongly shaped by the tumor microenvironment (TME), where malignant and non-malignant cells interact continuously. Single-cell approaches allow these interactions and cellular states to be examined at high resolution (Zhang et al., 2021). In this context, high-dimensional weighted gene co-expression network analysis (hdWGCNA) extends traditional WGCNA to scRNA-seq data, enabling the identification of coordinated gene modules that characterize specific tumor states (Morabito et al., 2023). Meanwhile, the growing availability of multi-omics resources makes it possible to test whether expression changes are genetically driven by leveraging expression quantitative trait loci (eQTLs) and Mendelian randomization (MR) (Liu et al., 2020; Li et al., 2023). Genome-wide association studies (GWAS) further provide large, diverse cohorts that support population-level causal inference. Combining these analytical layers with experimental validation can strengthen the discovery of clinically relevant genes.

As the prognosis of clear cell renal carcinoma is closely related to its clinical progression, and with the increasing availability of single-cell public data, we are delighted to find that existing ccRCC samples are well-equipped with comprehensive clinical information, particularly including clinical staging. This study aims to leverage single-cell samples integrated with clinical data, employing the hdWGCNA algorithm to identify clinically relevant module genomes. Subsequently, we plan to utilize expression quantitative trait loci (eQTL) data for Mendelian randomization. Finally, by combining next-generation sequencing and clinical data from The Cancer Genome Atlas (TCGA) and the Gene Expression Omnibus (GEO) databases, we will conduct a rigorous screening process in hopes of discovering new genes associated with the progression of clear cell renal carcinoma. This could potentially provide new therapeutic targets for the clinical treatment of ccRCC.

## Materials and Methods

### Data collection

Public scRNA-seq datasets were downloaded from the Gene Expression Omnibus (GEO). Summary-level MR exposure and eQTL data were obtained from the UK Biobank GWAS repository. Bulk RNA-seq profiles of ccRCC together with matched clinical annotations were retrieved *via* the UCSC Xena platform. Expression matrices were converted to transcripts per million (TPM) units and uniformly normalized to ensure comparability across cohorts.

### Processing and annotation of single-cell dataset

Initial single-cell preprocessing was performed with Seurat, including filtering, normalization, feature selection, scaling, and dimensionality reduction (Slovin et al., 2021). To mitigate batch effects across samples, Harmony was applied for integration (Korsunsky et al., 2019). Cell identity labels were assigned using SingleR, allowing systematic annotation of major immune and stromal compartments as well as malignant epithelial populations (Huang et al., 2021).

### Extraction of epithelial cells and hdWGCNA

CcRCC primarily originates from renal tubular epithelial cells. In the single-cell dataset, epithelial cells are considered as tumor cells. To isolate these epithelial cells, the ’subset’ function is applied. Subsequently, the hdWGCNA approach is employed to uncover modular gene patterns within the single-cell data. This selection is based on the ranking of module eigengene connectivity and practical considerations. Moreover, the analysis includes pseudo-temporal analysis based on the monocle2 package, which explores the developmental sequence of these cells (Hong et al., 2022). The communication patterns between cells are evaluated using the CellChat package, providing insights into intercellular interactions (Jin et al., 2021).

### hdWGCNA genes mendelian randomization analysis

Following module identification using hdWGCNA, we extracted the top 50 genes from each module. Genes were ranked by module eigengene connectivity (module membership; kME) to prioritize hub-like genes with maximal module membership. The “top 50” cutoff was selected as a literature-supported, commonly used scale for defining module core/hub gene sets and for network visualization/interpretation, while remaining practical for downstream MR instrument curation given finite eQTL coverage. Similar top-50 hub/core-gene selections have been used in prior WGCNA/hdWGCNA studies and protocols (Morabito et al., 2023; Backman et al., 2021). Instruments were selected as independent lead single-nucleotide polymorphisms (SNPs) after linkage disequilibrium (LD) clumping (*e.g.*, *r*^2^ < 0.001 within 10,000 kb using a European reference panel), retaining variants with genome-wide significant eQTL evidence where available and excluding weak instruments (mean F-statistic > 10 reported) (Zhou et al., 2019). Exposure and outcome datasets (both from UK Biobank) were harmonized to the effect allele, with strand-ambiguous palindromic SNPs removed or resolved by allele frequency; when necessary, proxy SNPs (*r*^2^ > 0.8) were used. Two-sample MR was performed in the TwoSampleMR package, using inverse-variance weighted (IVW) as the primary estimator and MR-Egger, weighted median, and weighted mode as sensitivity analyses, which aims to establish causal relationships between eQTLs and outcome exposures (Zhou et al., 2019). To evaluate whether hdWGCNA-identified genes were associated with patient outcomes, we retrieved and curated ccRCC bulk RNA-seq expression profiles and matched clinical survival data from the UCSC Xena portal. Survival-related candidates were screened using univariable Cox proportional hazards regression, with *p* < 0.05 considered statistically significant. Protein–protein interaction networks for these genes were obtained from the STRING database. Kaplan–Meier and related survival analyses were performed using the Human Protein Atlas online platform (https://www.proteinatlas.org).

### The immunological microenvironment analysis of the identified genes

The single-cell data for the validation dataset is compiled from the GEO database. Data organization and quality control are conducted using the ‘Seurat’ package and the SingleR’ package. Furthermore, the analysis encompasses cell-to-cell interactions, which are examined through the CellChat package, shedding light on cellular communication networks.

### Small interfering RNA (siRNA) transfection

The small interfering RNA (siRNA) was purchased from Nanjing Genlin Biological Technology Co., Ltd. The siPRDX2 sequence used in the experiment is as follows:5′-GCCUGGCAGUGACACGAUUAATT-3′, 5′-UUAAUCGUGUCACUGCCAGGCTT-3′. The siRNA transfection was carried out using Lipofectamine 3000 and Opti-MEM medium when the cell density was approximately 50%. RNA extraction or cellular functional experiments were conducted 48 h post-transfection

### Quantitative real-time polymerase chain reaction

Following the instructions of the reverse transcription kit (Vazyme, #R333), complementary DNA (cDNA) was obtained for quantitative real-time polymerase chain reaction (qRT-PCR). 2 ×ChamQ SYBR qPCR Master Mix was used as a fluorescent dye. Several sequences were utilized: PRDX2 forward primer ‘5-GTGTCCTTCGCCAGATCACT-3’ and reverse primer ‘5-ACGTTGGGCTTAATCGTGT-3’; beta-actin (β-actin) forward primer ‘5-ACTGGAACGGTGAAGGTGAC-3’,and reverse primer ‘5-AGAGAAGTGGGGTGGCTTTT-3’.

### Cell counting kit-8 assay, clone formation assay and transwell assay

Following established protocols, cell proliferation was evaluated using Cell Counting Kit-8 (CCK-8) and colony formation assays. After siRNA transfection, 786-O and Caki-1 cells were maintained in a Thermo Fisher Scientific incubator and then plated into 96-well plates at a density of 1,500 cells per well. CCK-8 reagent (10 μl) was introduced into each well at 24, 48, 72, and 96 h post-seeding. Plates were gently agitated to ensure uniform mixing and incubated for an additional 1.5 h. Optical density at 450 nm (OD450) was recorded with a microplate reader. For the colony formation experiment, 1,500 cells were seeded per well in six-well plates. Once colonies became visible, cells were fixed with formaldehyde and stained with crystal violet, after which colony growth was documented. Cell migration was examined by Transwell assays. Serum-free cell suspensions were placed in the upper chambers, while the lower chambers contained medium supplemented with 10% fetal bovine serum. After incubation, cells remaining on the upper surface were removed, whereas migrated cells on the underside were fixed, stained, and quantified.

## Results

### Data collection

We obtained information from six ccRCC patients from the GSE156632 dataset. SNP data was downloaded from the GWAS database, with the dataset named ukb-b-1316. Transcriptome data was acquired from TCGA-KIRC, organized in TPM format, and standardized. Additionally, clinical information of corresponding patients from TCGA-KIRC was curated. For validation, single-cell data was sourced from GSE159115, encompassing a total of seven ccRCC datasets.The clinical grouping information for the single-cell data and clinical information for TCGA patients can be found in the supplementary materials.

### Data preprocessing and annotation of single-cell data

**Figure 1:**
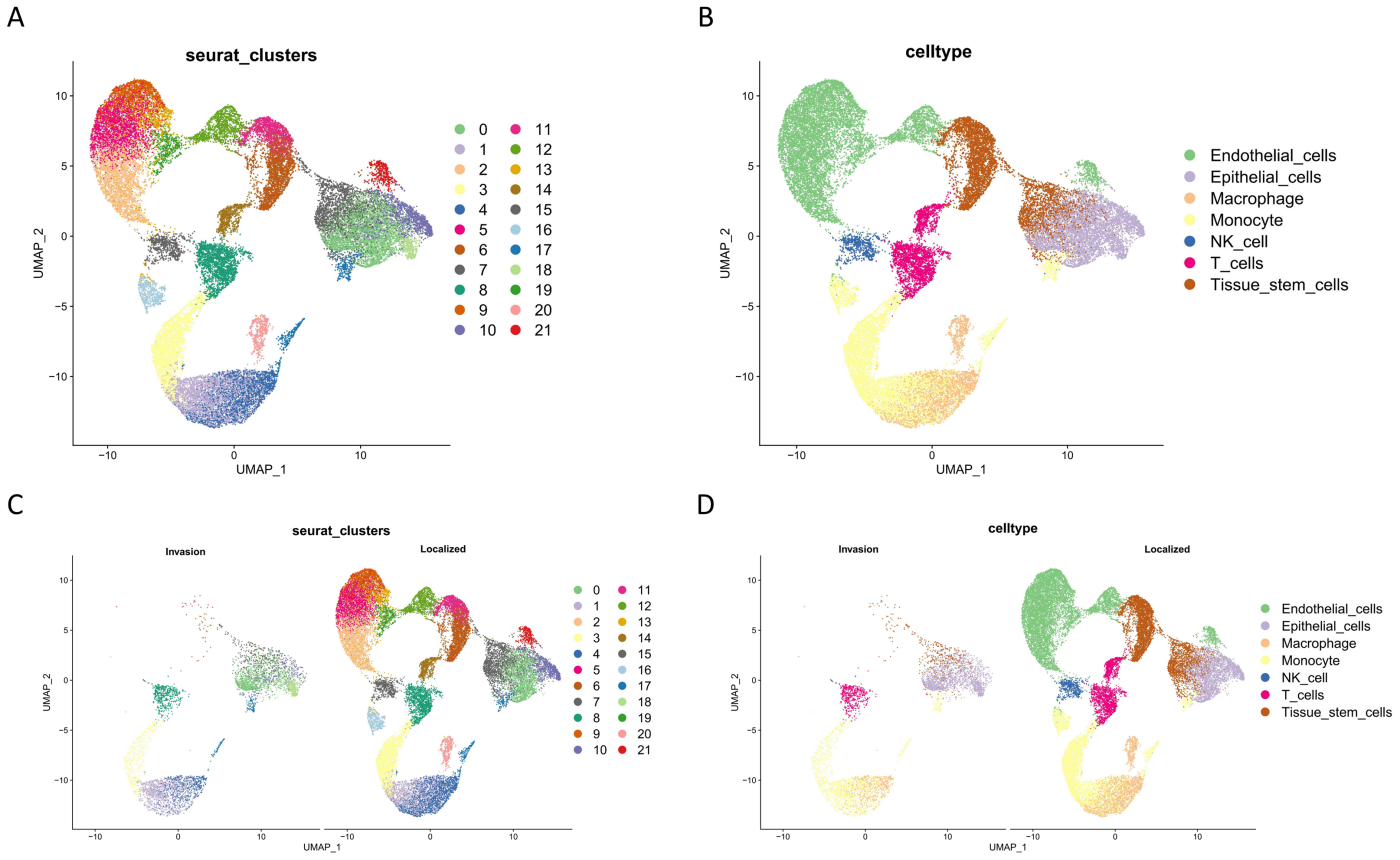
Single-cell data analysis. (A) Cell clustering; (B) cell type annotation; (C) cell clustering displayed by group; (D) cell type annotation displayed by group.

We integrated the GSE156632 dataset using the Seurat package. The data was standardized, high-variability genes were identified, data was centered, and principal component analysis (PCA) was performed using functions NormalizeData, FindVariableFeatures, ScaleData, and RunPCA. Subsequently, the data was batch-corrected using Harmony. Finally, dimensionality reduction was performed using uniform manifold approximation and projection (UMAP) *via* RunUMAP, resulting in the identification of 22 cell subpopulations (Figs. 1A, 1C). SingleR package was then used to annotate each cell subpopulation. Ultimately, seven distinct cell clusters were identified, including endothelial cells, epithelial cells, macrophages, monocytes, NK cells, T cells, and tissue stem cells (Figs. 1B, 1D). The cells were further grouped based on staging (T2 as Localized, T3 as Invasion), and considering that ccRCC originates from renal tubular epithelial cells, we classified Epithelial cells as tumor cells.

### Extraction of epithelial cells and hdWGCNA

**Figure 2:**
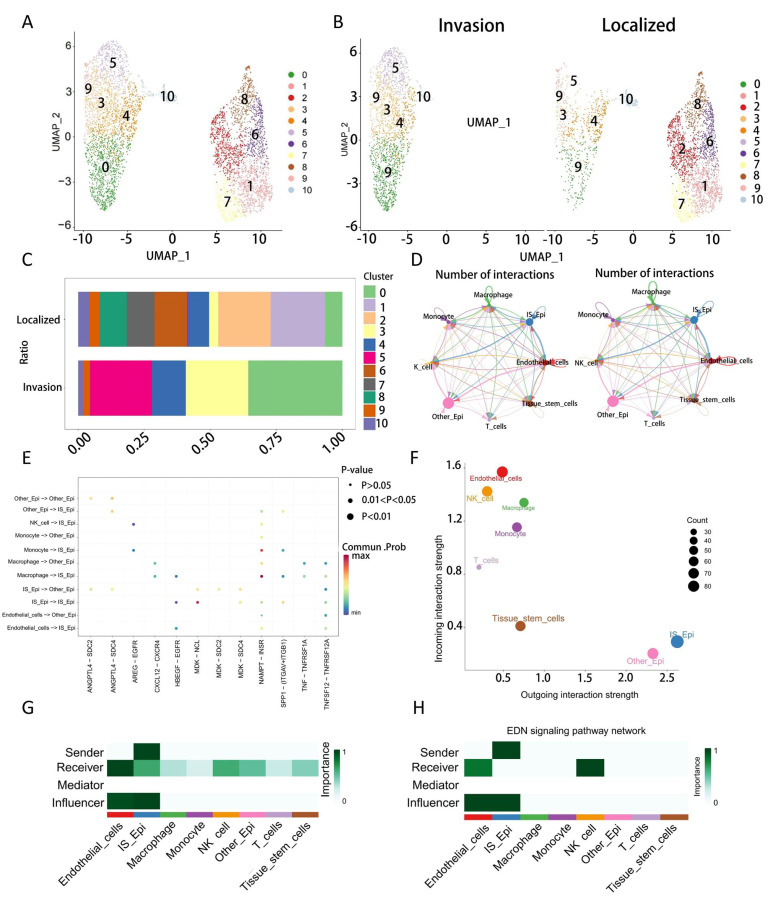
Epithelial cell subgroup selection. (A) Epithelial cell clustering. (B) Epithelial cell clustering displayed by clinical group. (C) Cell group proportions. (D) Overview of cell communication. (E) Cell communication bubble plot. (F) Cell communication numbers. (G, H) MK, EDN signaling pathway.

**Figure 3:**
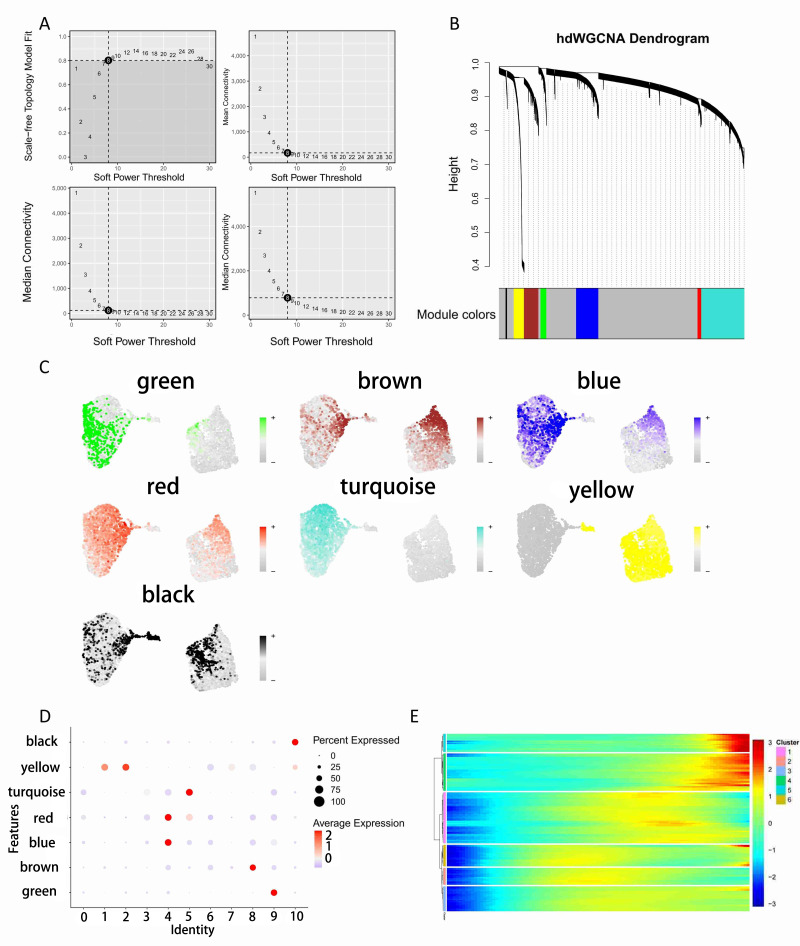
HdWGCNA analysis. (A) hdWGCNA SoftPower selection. (B) hdWGCNA dendrogram. (C) Expression of each module in single-cell data. (D) Expression of module genes across clusters. (E) Number and weight of cellular communications.

We isolated the epithelial cell population and grouped them into T2 (Localized) and T3 (Invasion) stages (Figs. 2A, 2B). Subsequently, we performed dimensionality reduction clustering on these epithelial cells and identified that subpopulations 0, 3, and 5 were more predominant in the Invasion group (Fig. 2C). To investigate the potential differences in cell communication between these three subpopulations and the others, we divided the epithelial cells into two groups: invasion-stage epithelial cells (IS_Epi; clusters 0, 3, and 5) and other epithelial cells (Other_Epi). Next, we randomly selected 2,000 cells for cell communication analysis and observed significant differences in communication levels between cell subpopulations (Fig. 2D). The interaction analysis revealed that IS_Epi group exhibited more frequent interactions with endothelial cells and had a higher total communication count compared to Other_Epi group. This indicates that IS_Epi cells engage in more frequent interactions with endothelial cells (Figs. 2E and 2F, 2G, 2H). Considering the close association between the staging of renal cancer and vascular invasion, and the higher representation of IS_Epi group in the invasive group, we selected IS_Epi group as the target cell group for hdWGCNA.Following this, we conducted hdWGCNA analysis with the goal of identifying co-expressed gene modules, resulting in the discovery of seven distinct modules (Figs. 3A, 3B). We then selected modules that were specifically expressed in subpopulations 0, 3, and 5, which were turquoise, red, and blue modules, comprising a total of 150 genes (Figs. 3C, 3D). Subsequently, we performed pseudotime analysis on these 150 genes and observed that these genes were predominantly distributed in both early and late stages of cell differentiation (Fig. 3E).

### Mendelian randomization analysis of hdWGCNA genes

**Figure 4:**
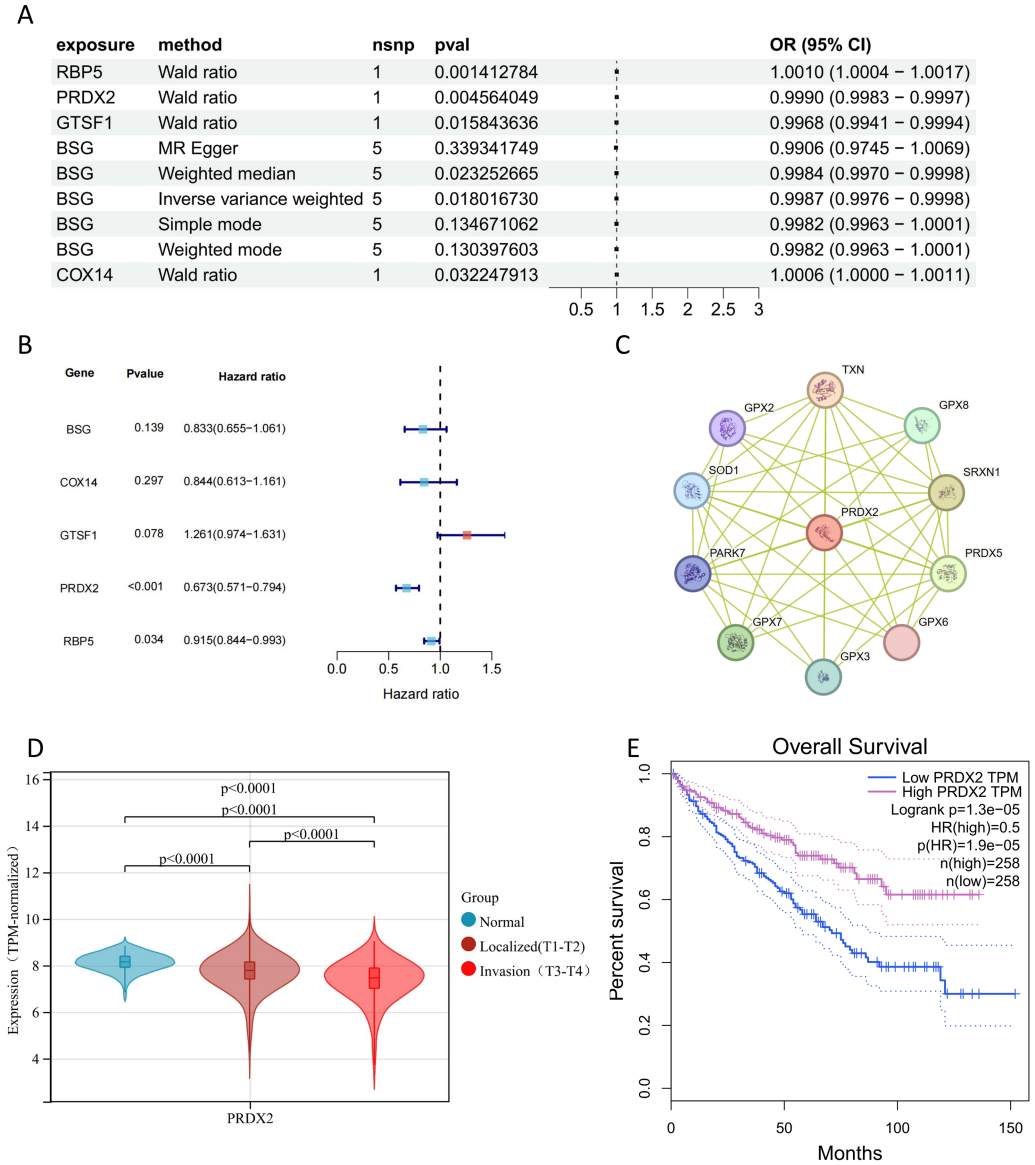
Identification of key insights. (A) Mendelian randomization results. (B) Univariate cox regression of mendelian randomization results. (C) PRDX2 protein interaction network. (D) Expression of PRDX2 in different clinical groups. (E) Impact of PRDX2 gene on patient survival.

After extracting the relevant genes, we obtained their eQTL information from the gwas database and identified the exposure outcome data (ukb-b-1316). Following Mendelian randomization analysis, we found that genes such as RBP5, PRDX2, GTSF1, BSG, and COX14 had a causal relationship with the exposure outcome (Fig. 4A). Because the genes we identified had relatively small odds ratio (OR), we suspect that this could be due to the limited number of outcome cases compared to the control cases in ukb-b-1316. The relatively narrow confidence intervals likely reflect the precision of the UK Biobank outcome GWAS estimates, which—despite a modest number of kidney cancer cases (*n* = 1,114)—included a very large control set (*n* = 461,896; total *n* = 463,010), yielding stable SNP–outcome association estimates. To address this concern, we downloaded data from the TCGA-KIRC database, integrated clinical information of patients, and conducted a univariate cox regression analysis. In this analysis, we found that both PRDX2 and RBP5 had a significant impact on patient prognosis (Fig. 4B). Notably, while RPB5 showed an inconsistent relationship with patient prognosis between the Mendelian randomization results and the univariate Cox regression analysis, PRDX2 consistently exhibited a positive influence on patient prognosis in both the univariate cox regression and Mendelian randomization studies. High expression of PRDX2 consistently indicated a better prognosis for patients.Therefore, we selected PRDX2 for further analysis. Utilizing the STRING database, we conducted an analysis of proteins associated with PRDX2 (Fig. 4C). Additionally, we examined the expression of PRDX2 in normal tissues, localized tumors, and invasive tumors (Fig. 4D). This examination revealed a gradual decrease in PRDX2 expression with the increasing aggressiveness of tumors. Furthermore, survival analysis demonstrated that in clear cell renal cell carcinoma, patients with high PRDX2 expression levels experienced significantly better prognoses (Fig. 4E).

**Figure 5:**
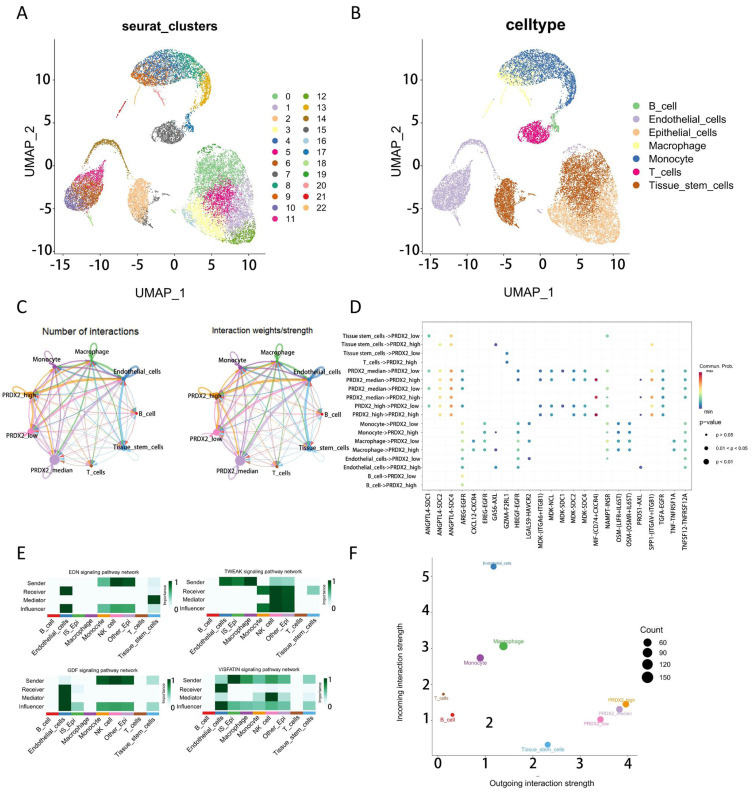
The impact of PRDX2 gene on cellular communication. (A) Cell clustering. (B) Cell type annotation. (C) Overview of cellular communication. (D) Bubble plot of cellular communication overview. (E) Influence of PRDX2 expression on signaling pathways such as EDN, TWEAK, GDF, and VISFATIN. (F) Number and weight of cellular communications.

### The immunological microenvironment analysis of the identified genes

We have identified PRDX2 as having a significant impact on the prognosis of ccRCC, with its high expression potentially indicating a better prognosis for patients. Subsequently, we downloaded single-cell data from GSE159115 and performed cell clustering and cell type identification (Figs. 5A, 5B). Based on the expression of PRDX2 in tumor cells (epithelial cells), we divided the epithelial cells into three groups using quartiles (Figs. 5C, 5D): PRDX2low (PRDX2 expression below the 25th percentile), PRDX2median (PRDX2 expression between the 25th and 75th percentiles), and PRDX2high (PRDX2 expression above the 75th percentile). While, overall, the pathway intensity in the PRDX2-low group is lower than that in the PRDX2-high group (Fig. 5F), the cellular communication level between PRDX2-low and vascular endothelial cells is more intimate. Clinical staging of ccRCC often depends on whether there is invasion of surrounding blood vessels and tissues. Therefore, PRDX2 may potentially influence the migration of ccRCC cells in the tumor microenvironment, subsequently impacting patient prognosis. We observed their interactions with other cells in the tumor microenvironment, and we found that as the expression of PRDX2 changed, the strength of interaction between epithelial cells and other cells in the tumor microenvironment also varied. Furthermore, we discovered that in the EDN, TWEAK, GDF, and VISFATIN signaling pathways (pathway abbreviations as defined by CellChat), the PRDX2low group exhibited significantly stronger interactions with endothelial cells compared to the PRDX2high group (Fig. 5E).

### Cell culture and clinical sample validation

**Figure 6:**
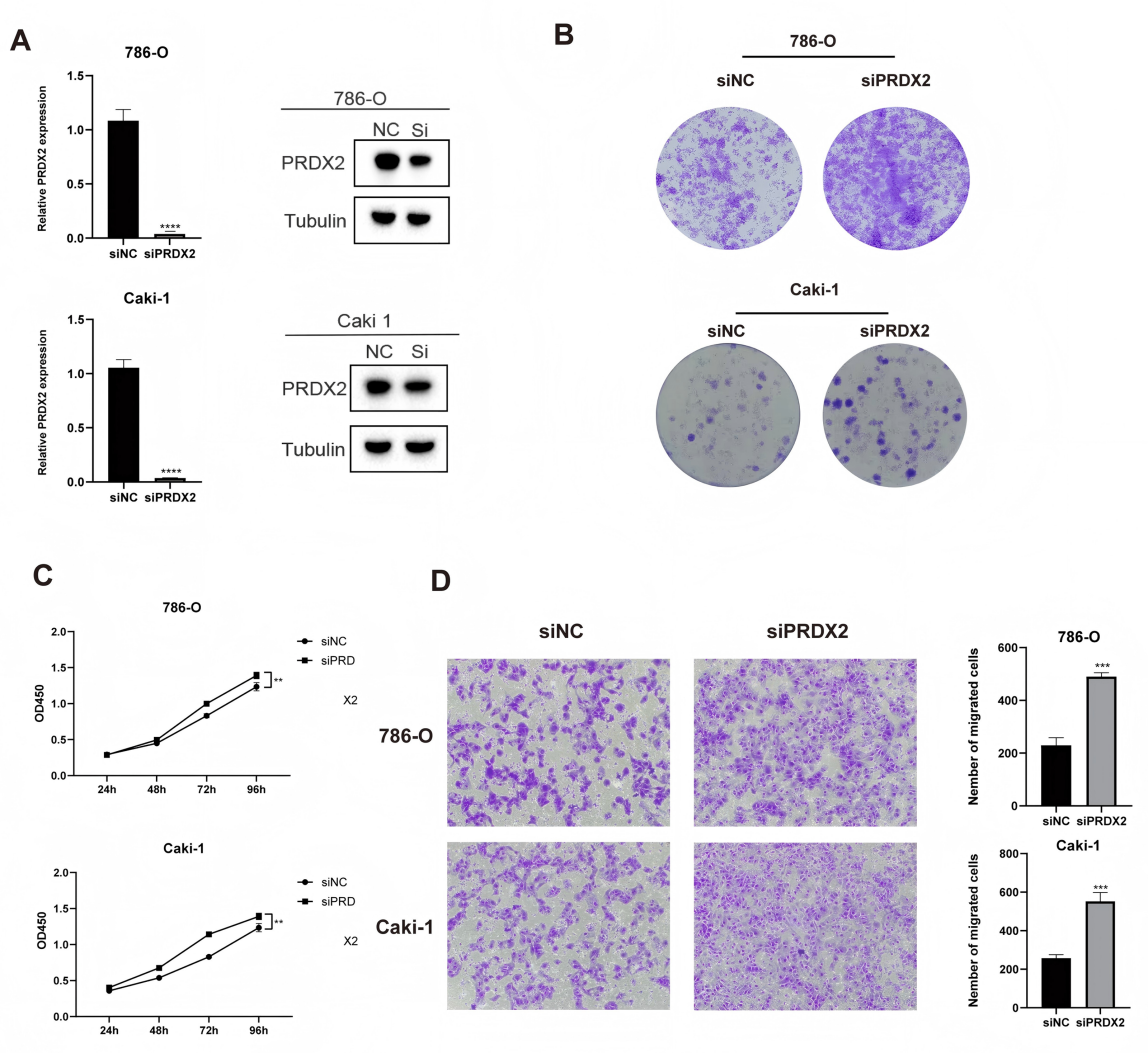
Cellular experiments. (A) qRT-PCR and Western Blot show the knockdown efficiency of PRDX2, (B) colony formation assay and (C) CCK8 assay indicate enhanced 786-O and Caki-1 cells proliferation ability after PRDX2 knockdown; (D) PRDX2 knockdown promotes ccRCC cells migration ability in 786-O and Caki-1 cells.

In different single-cell datasets, PRDX2 seems to have an impact on the interaction between ccRCC cells and vascular endothelial cells, as well as the tumor microenvironment. Furthermore, the results of next-generation sequencing and Mendelian randomization suggest that PRDX2 appears to be a protective factor for ccRCC patients. Therefore, we aim to observe the influence of PRDX2 on ccRCC at the cellular level. ccRCC cell lines 786-O and Caki-1 were used for subsequent cellular functional experiments. We first transfected siPRDX2 into 786-O and Caki-1 cells to knock down PRDX2. The results indicated that siPRDX2 used in the experiment effectively inhibited the expression of PRDX2 in 786-O and Caki-1 cells (Fig. 6A). Cell Counting Kit-8 (CCK8) and colony formation assays demonstrated that PRDX2 knockdown significantly promoted the proliferation of 786-O and Caki-1 cells (Figs. 6B, 6C). Transwell assay showed that 786-O and Caki-1 cells treated with siPRDX2 exhibited higher migration ability compared to the negative control group (Fig. 6D). This is consistent with our previous analysis, suggesting that downregulation of PRDX2 plays a role in promoting the proliferation and migration of ccRCC cells.

## Discusion

Our study took a clinical perspective, utilizing clinical information for grouping in the single-cell dataset. We employed hdWGCNA to select modular genes, with the core focus of identifying cell subpopulations associated with later clinical staging and higher tumor invasiveness. Traditional single-cell sequencing data analysis often relies on differential analysis for gene selection. However, single-cell data is characterized by shallow sequencing depth and limited breadth. Moreover, differential genes in single-cell sequencing data often rely on average values for calculation, which may not be as effective as traditional bulk sequencing in identifying disease-related genes due to the larger sample sizes in the latter. This is evident when certain genes showing significant differences in bulk sequencing data do not display differential expression in single-cell sequencing data. However, the advantage of single-cell sequencing lies in its cell-based resolution, providing better insights into cellular interactions within the tumor microenvironment.Another important consideration is that when comparing tumors to each other, as opposed to comparing tumors to normal tissues, the differences are often smaller. Therefore, our research strategy focuses on leveraging the high resolution of single-cell sequencing to identify cell subpopulations with significantly different expression profiles among various clinical groups. We then use hdWGCNA to select genes that are differentially expressed in these subpopulations. These genes can be linked to clinical outcomes by exploiting data from the GWAS database, which, like single-cell data, involves a large number of cases. However, the GWAS database may lack the granularity needed to precisely classify diseases, such as the TNM staging and specific survival information of patients.

Following Mendelian randomization analysis, we conducted further gene analysis using data from TCGA. This led us to identify PRDX2 as a gene that appears to be associated with the development of ccRCC and may serve as a protective factor for the disease. This is indicated by the differences in its expression between normal tissues and tumor tissues, as well as between low- and high-stage malignant tumor tissues. Bulk sequencing data strongly confirmed this finding, showing a gradual decrease in PRDX2 expression with increasing malignancy. Additionally, survival analysis indicated that patients with high PRDX2 expression had better prognoses, consistent with Mendelian randomization’s causal inference. Subsequent cytological experiments have confirmed that the knockdown of PRDX2 could increase the proliferation and migration capabilities of ccRCC cells. Although PRDX2 was efficiently knocked down (Fig. 6A), the CCK-8 readout (OD450) showed only modest differences (Fig. 6C). This is expected because OD450 primarily reflects metabolic activity/viability at fixed time points and may be relatively insensitive to subtle changes in proliferation rate over short intervals. This aligns precisely with our analysis, implying that the expression of PRDX2 influences the migration ability of ccRCC cells, coinciding with the initial focus of our research. Simultaneously, this also indicates that PRDX2 is highly likely to be a potential target and prognostic factor for ccRCC.

PRDX2 encodes a member of the peroxiredoxin antioxidant enzyme family, capable of reducing hydrogen peroxide and alkyl hydroperoxides. The encoded protein may play an antioxidant protective role in cells. In existing studies, PRDX2 has been found to play a role in the progression of various epithelial-derived tumors. For example, in gastric cancer, the knockdown of PRDX2 leads to increased levels of ROS (reactive oxygen species), oxidative DNA damage, and double-strand DNA breaks, while significantly sensitizing gastric cancer cells to cisplatin treatment (Wang et al., 2020). In colorectal cancer, PRDX2 has been found to promote the proliferation of colorectal cancer cells by increasing the ubiquitination-mediated degradation of p53, leading to poor prognosis (Wang et al., 2021). Additionally, novel drug research has found that targeting PRDX2 directly induces ROS-mediated apoptosis in colorectal cancer cells (Lv et al., 2023). In current studies, clear cell renal carcinoma has been found to be accompanied by reprogramming of glucose and fatty acid metabolism as well as the tricarboxylic acid cycle. The metabolism of tryptophan, arginine, and glutamine has also been found to be reprogrammed in many ccRCC patients (Zeng et al., 2023; Wettersten et al., 2017). Cell death, including various forms such as apoptosis, necrosis, and pyroptosis, plays a critical role in tumor initiation and progression (Pan et al., 2022; Chakiryan et al., 2023). In ccRCC, the regulation of cell death pathways can influence tumor growth, immune evasion, and resistance to therapy (Chen et al., 2024). Specifically, pyroptosis, a form of programmed cell death driven by inflammasomes, has been shown to correlate with the tumor microenvironment, affecting both immune infiltration and angiogenesis (Park et al., 2016). The balance of cell death and survival pathways, including the modulation of redox signaling by PRDX2, is crucial in ccRCC progression and may offer therapeutic insights for targeting these processes (Kim & Jang, 2019; Soini et al., 2006; Zhu et al., 2023). In ccRCC, pseudohypoxia downstream of von Hippel–Lindau/hypoxia-inducible factor (VHL/HIF) is coupled to glycolytic partitioning, attenuated oxidative phosphorylation (OxPhos), and remodeled lipid metabolism, collectively constraining reactive oxygen species (ROS) production (Bacigalupa & Rathmell, 2020). Consistent with ccRCC’s highly immune-infiltrated TME, PRDX2-low tumor states showed stronger endothelial and inflammatory ligand–receptor signaling, aligning with angiogenic and immune-dysregulated contexts. Given that redox metabolism shapes cytokine production, antigen processing, and myeloid/T-cell function, we hypothesize that PRDX2 constrains immunophlogosis; reduced PRDX2 may favor pro-angiogenic, inflamed yet functionally exhausted niches with therapeutic relevance (Salzano et al., 2014; Monjaras-Avila et al., 2023). In addition to its critical role in metabolic reprogramming, PRDX2 is increasingly recognized as a modulator of the immune landscape in ccRCC (Xia et al., 2023). The tumor microenvironment (TME) of ccRCC is highly immune-infiltrated, and recent studies suggest that redox signaling, particularly through enzymes like PRDX2, influences immune cell behavior and cytokine production (Rhee, 2016). PRDX2 regulates the balance between oxidative and reductive stress in immune cells, which can alter macrophage polarization and T-cell function, key components of the TME (Sadowska-Bartosz & Bartosz, 2023; Harrison, Zinovkin & Pranjol, 2024). In PRDX2-low states, heightened ROS levels lead to the activation of pro-inflammatory signaling pathways, including those involved in angiogenesis and immune dysregulation, such as EDN, TWEAK, and VISFATIN (Dawas et al., 1999). This exacerbates the inflammatory microenvironment, promoting tumor progression by fostering an immune-suppressive niche that supports angiogenesis and evasion of immune surveillance (Liu, Xiao & Xia, 2017; Heravi et al., 2022). Furthermore, PRDX2’s role in regulating lipid metabolism is of particular interest. Lipid accumulation and altered fatty acid oxidation are hallmarks of ccRCC, and PRDX2 may play a role in maintaining lipid homeostasis within the TME. By modulating oxidative stress and lipid peroxidation, PRDX2 could influence the activation of key metabolic and inflammatory pathways involved in ccRCC progression (Krishna et al., 2021). Overall, the dual role of PRDX2 in both metabolic regulation and immune modulation underscores its potential as a therapeutic target in ccRCC, where restoring redox balance could improve the efficacy of immune and metabolic-based therapies (Harrison, Zinovkin & Pranjol, 2024; Krishna et al., 2021).

In our study, various bioinformatics techniques found that PRDX2 is associated with the development and progression of ccRCC, and *in vitro* experiments showed that knocking down PRDX2 inhibits the proliferation of ccRCC cells. Considering the metabolic reprogramming of clear cell renal carcinoma and the role of PRDX2 in altering the migration and proliferation of various cancers through metabolic influence, we hypothesize that PRDX2 may influence the proliferation and migration of ccRCC. Additionally, ccRCC has been identified as a highly immune-infiltrated tumor (Hu et al., 2020) with varying immune landscapes among different patients (Hu et al., 2020; Shi et al., 2022). Our research indicates that there are significant changes in communication between ccRCC cells and immune cells within groups with high and low PRDX2 expression. This suggests that PRDX2 may also influence the immune landscape of ccRCC. Therefore, given that the core feature of ccRCC is metabolic reprogramming, PRDX2 influences cellular metabolism in various cancers, and our study shows it affects the proliferation and migration of clear cell renal carcinoma, while also being an immune-infiltrated tumor where PRDX2 expression affects the immune landscape according to single-cell data, we hypothesize that PRDX2 may be a factor linking metabolic reprogramming in ccRCC. Can it connect metabolic reprogramming with the immune landscape in ccRCC? This hypothesis is the core direction we need to explore in our subsequent research.

In conclusion, our analysis suggests that PRDX2 may be linked to the development and progression of ccRCC. The expression level of PRDX2 may serve as a prognostic indicator for patients. Moreover, PRDX2 may influence cell interactions in the tumor microenvironment. Our analysis revealed that the level of communication between PRDX2 low-expression groups and endothelial cells was significantly higher in signaling pathways such as EDN, TWEAK, GDF, and VISFATIN. Since vascular invasion is an important indicator of clinical staging in ccRCC (Stresing et al., 2013), the low-expression group of PRDX2 cancer cells may be more prone to vascular invasion, implying later staging and poorer prognosis. This observation aligns with our analytical results. Furthermore, while many studies suggest that PRDX2 acts as a cancer-promoting gene in epithelial-origin tumors such as colorectal and breast cancer (Wang et al., 2021; Chen et al., 2024), our findings in ccRCC seem to indicate that it may be a tumor-suppressing gene. Other studies have also found that PRDX2 plays a tumor-suppressing role in ccRCC, but our evidence is more substantial (Ren et al., 2021). While other studies are entirely based on databases and rely on a single source of evidence, our research is the first to conduct experimental studies on the PRDX2 gene in ccRCC, utilizing diverse data sources. Our study not only involves single-cell genomics and traditional bulk-seq but also incorporates GWAS data. However, due to budget and time constraints, our study lacks some clinical sample data and depth. At the same time, our study will also draw on existing well-established research, such as the work by Meng et al., who recently delineated a Multi-omic Subtype (MoS) classification, identifying three distinct subtypes: immune “cold”, immune “hot”. This work has been highly inspirational for us. Our research primarily focuses on the identification of individual molecules, whereas Meng et al.’s study places greater emphasis on patient prognosis and precision therapy (Meng et al., 2023; Yin et al., 2023). Therefore, we plan to delve deeper into the underlying mechanisms of PRDX2’s impact on patients in future research and supplement our observations of the precise effects of PRDX2 on patient prognosis with large-scale clinical data.

## Conclusion

In this study, we utilized single-cell data in combination with hdWGCNA and Mendelian randomization to identify genes associated with ccRCC. Further validation was performed using transcriptome data. Additionally, experimental evidence demonstrated that knockdown of PRDX2 may enhance the proliferation and migration of 786-O and Caki-1 cells, suggesting a potential therapeutic target for ccRCC.

## Supplemental Information

10.7717/peerj.21057/supp-1 Supplemental Information 1 Cell migration and colony formation experiments in 786 cells

10.7717/peerj.21057/supp-2 Supplemental Information 2 Cell migration and colony formation experiments in Caki-1 cells

10.7717/peerj.21057/supp-3 Supplemental Information 3 Code

10.7717/peerj.21057/supp-4 Supplemental Information 4 MIQE checklist

10.7717/peerj.21057/supp-5 Supplemental Information 5 Western bolt and cell colon experience

10.7717/peerj.21057/supp-6 Supplemental Information 6 STROBE Checklist

## Additional Information and Declarations

### Data Availability

The following information was supplied regarding data availability: The raw data are available in the Supplemental Files. The single cell sequencing data is available in GEO: GSE156632 and GSE159115. The GWAS summary data are available in IEU OPEN GWAS PROJECT: ukb-b-1316. The bulk sequencing data and clinical information are available in TCGA: TCGA-KIRC.
