# Supplementary material for: Multi-omics analysis reveals key genes associated with clear cell renal cell carcinoma
Source: PeerJ. 2026 Apr 21;14:e21057. doi: 10.7717/peerj.21057 (PMC13108463; doi:10.7717/peerj.21057)
Supplement: Supplemental Information 6 [file peerj-14-21057-s006.docx]

**STROBE-MR checklist of recommended items to address in reports of Mendelian randomization studies**^1^ ^2^

| **Item No.** | **Section** | **Checklist item** | **Page No.** | **Relevant text from manuscript** |
| --- | --- | --- | --- | --- |
| 1 | **TITLE and ABSTRACT** | Indicate Mendelian randomization (MR) as the study’s design in the title and/or the abstract if that is a main purpose of the study | Title Page, Abstract | Mentioned in Abstract: 'Mendelian randomization analysis' |
|  | **INTRODUCTION** |  |  |  |
| 2 | **Background** | Explain the scientific background and rationale for the reported study. What is the exposure? Is a potential causal relationship between exposure and outcome plausible? Justify why MR is a helpful method to address the study question | Introduction | Explained: ccRCC staging, prognosis, MR necessity |
| 3 | **Objectives** | State specific objectives clearly, including pre-specified causal hypotheses (if any). State that MR is a method that, under specific assumptions, intends to estimate causal effects | Introduction | Clear objective to identify prognosis-related genes using MR |
|  | **METHODS** |  |  |  |
| 4 | **Study design and data sources** | Present key elements of the study design early in the article. Consider including a table listing sources of data for all phases of the study. For each data source contributing to the analysis, describe the following: | Data Availability Statement | Public datasets: The GWAS summary data that support the findings of this study are openly available in IEU OPEN GWAS PROJECT at [https://gwas.mrcieu.ac.uk/], reference number [ukb-b-1316] |
|  | a) | Setting: Describe the study design and the underlying population, if possible. Describe the setting, locations, and relevant dates, including periods of recruitment, exposure, follow-up, and data collection, when available. | METHODS | Public datasets: The GWAS summary data that support the findings of this study are openly available in IEU OPEN GWAS PROJECT at [https://gwas.mrcieu.ac.uk/], reference number [ukb-b-1316] |
|  | b) | Participants: Give the eligibility criteria, and the sources and methods of selection of participants. Report the sample size, and whether any power or sample size calculations were carried out prior to the main analysis | METHODS | Yes |
|  | c) | Describe measurement, quality control and selection of genetic variants | METHODS | Yes，In the conde |
|  | d) | For each exposure, outcome, and other relevant variables, describe methods of assessment and diagnostic criteria for diseases | METHODS | Yes，In the conde |
|  | e) | Provide details of ethics committee approval and participant informed consent, if relevant | No | The cell lines used in our study were obtained from the Cell Bank of the Chinese Academy of Sciences. Ethical approval for studies involving human subjects was not required |
| 5 | **Assumptions** | Explicitly state the three core IV assumptions for the main analysis (relevance, independence and exclusion restriction) as well assumptions for any additional or sensitivity analysis | No | Only used for simple filtering |
| 6 | **Statistical methods: main analysis** | Describe statistical methods and statistics used |  |  |
|  | a) | Describe how quantitative variables were handled in the analyses (i.e., scale, units, model) | METHODS | TPM format for expression data |
|  | b) | Describe how genetic variants were handled in the analyses and, if applicable, how their weights were selected | METHODS | Top 50 hdWGCNA genes used |
|  | c) | Describe the MR estimator (e.g. two-stage least squares, Wald ratio) and related statistics. Detail the included covariates and, in case of two-sample MR, whether the same covariate set was used for adjustment in the two samples | METHODS | TwoSampleMR package used |
|  | d) | Explain how missing data were addressed | METHODS | Recommend adding clarification if needed |
|  | e) | If applicable, indicate how multiple testing was addressed | METHODS | Drafted in 'Additional Analytical Considerations' |
| 7 | **Assessment of assumptions** | Describe any methods or prior knowledge used to assess the assumptions or justify their validity | METHODS | No |
| 8 | **Sensitivity analyses and additional analyses** | Describe any sensitivity analyses or additional analyses performed (e.g. comparison of effect estimates from different approaches, independent replication, bias analytic techniques, validation of instruments, simulations) | METHODS | Yes，experimental validation |
| 9 | **Software and pre-registration** |  |  |  |
|  | a) | Name statistical software and package(s), including version and settings used | METHODS | TwoSampleMR，R |
|  | b) | State whether the study protocol and details were pre-registered (as well as when and where) | No | Just a simple screening |
|  | **RESULTS** |  |  |  |
| 10 | **Descriptive data** |  |  |  |
|  | a) | Report the numbers of individuals at each stage of included studies and reasons for exclusion. Consider use of a flow diagram | Results 3.1 | Stated; no flowchart provided |
|  | b) | Report summary statistics for phenotypic exposure(s), outcome(s), and other relevant variables (e.g. means, SDs, proportions) | Results 3.1-3.3 | Supplementary materials referenced |
|  | c) | If the data sources include meta-analyses of previous studies, provide the assessments of heterogeneity across these studies | N/A | Not applicable to study design |
|  | d) | For two-sample MR:  i.  Provide justification of the similarity of the genetic variant-exposure associations between the exposure and outcome samples  ii.  Provide information on the number of individuals who overlap between the exposure and outcome studies | Implied | Independent samples assumed; not explicitly stated |
| 11 | **Main results** |  |  |  |
|  | a) | Report the associations between genetic variant and exposure, and between genetic variant and outcome, preferably on an interpretable scale | Results 3.4 | Associations reported |
|  | b) | Report MR estimates of the relationship between exposure and outcome, and the measures of uncertainty from the MR analysis, on an interpretable scale, such as odds ratio or relative risk per SD difference | Results 3.4 | ORs and survival data provided |
|  | c) | If relevant, consider translating estimates of relative risk into absolute risk for a meaningful time period | Not performed | Could be considered in future studies |
|  | d) | Consider plots to visualize results (e.g. forest plot, scatterplot of associations between genetic variants and outcome versus between genetic variants and exposure) | Figures 4, 5 | Included: Forest plots, networks, survival curves |
| 12 | **Assessment of assumptions** |  |  |  |
|  | a) | Report the assessment of the validity of the assumptions | Discussion | Discussed imbalance; no formal tests |
|  | b) | Report any additional statistics (e.g., assessments of heterogeneity across genetic variants, such as *I^2^*, Q statistic or E-value) | Not performed | Recommend including in future studies |
| 13 | **Sensitivity analyses and additional analyses** |  |  |  |
|  | a) | Report any sensitivity analyses to assess the robustness of the main results to violations of the assumptions | Results 3.4 | Cox regression validation |
|  | b) | Report results from other sensitivity analyses or additional analyses | Results 3.5 | In vitro validation conducted |
|  | c) | Report any assessment of direction of causal relationship (e.g., bidirectional MR) | Not tested | Could add bidirectional MR if needed |
|  | d) | When relevant, report and compare with estimates from non-MR analyses | Results 3.4 | TCGA Cox regression compared |
|  | e) | Consider additional plots to visualize results (e.g., leave-one-out analyses) | Figures 4, 5 | Provided |
|  | **DISCUSSION** |  |  |  |
| 14 | **Key results** | Summarize key results with reference to study objectives | Discussion | PRDX2 identified, linked to clinical outcomes |
| 15 | **Limitations** | Discuss limitations of the study, taking into account the validity of the IV assumptions, other sources of potential bias, and imprecision. Discuss both direction and magnitude of any potential bias and any efforts to address them | Discussion | Bias, GWAS sample size, clinical data depth discussed |
| 16 | **Interpretation** |  | Discussion | Explained carefully |
|  | a) | Meaning: Give a cautious overall interpretation of results in the context of their limitations and in comparison with other studies | Discussion | PRDX2 role discussed |
|  | b) | Mechanism: Discuss underlying biological mechanisms that could drive a potential causal relationship between the investigated exposure and the outcome, and whether the gene-environment equivalence assumption is reasonable. Use causal language carefully, clarifying that IV estimates may provide causal effects only under certain assumptions | Discussion | Potential biomarker, therapeutic target |
|  | c) | Clinical relevance: Discuss whether the results have clinical or public policy relevance, and to what extent they inform effect sizes of possible interventions | Discussion | Explained carefully |
| 17 | **Generalizability** | Discuss the generalizability of the study results (a) to other populations, (b) across other exposure periods/timings, and (c) across other levels of exposure | Discussion | Need for larger validation stated |
|  | **OTHER INFORMATION** |  |  |  |
| 18 | **Funding** | Describe sources of funding and the role of funders in the present study and, if applicable, sources of funding for the databases and original study or studies on which the present study is based | Additional Supplement | No specific funding; internal support stated |
| 19 | **Data and data sharing** | Provide the data used to perform all analyses or report where and how the data can be accessed, and reference these sources in the article. Provide the statistical code needed to reproduce the results in the article, or report whether the code is publicly accessible and if so, where | Section 7 | Public data; code available upon request |
| 20 | **Conflicts of Interest** | All authors should declare all potential conflicts of interest | Section 8 | None declared |

This checklist is copyrighted by the Equator Network under the Creative Commons Attribution 3.0 Unported (CC BY 3.0) license.

1. Skrivankova VW, Richmond RC, Woolf BAR, Yarmolinsky J, Davies NM, Swanson SA, et al. Strengthening the Reporting of Observational Studies in Epidemiology using Mendelian Randomization (STROBE-MR) Statement. JAMA. 2021;under review.

2. Skrivankova VW, Richmond RC, Woolf BAR, Davies NM, Swanson SA, VanderWeele TJ, et al. Strengthening the Reporting of Observational Studies in Epidemiology using Mendelian Randomisation (STROBE-MR): Explanation and Elaboration. BMJ. 2021;375:n2233.
